# Therapeutic regulation of complement activation in extracorporeal circuits and intravascular treatments with special reference to the alternative pathway amplification loop

**DOI:** 10.1111/imr.13148

**Published:** 2022-10-18

**Authors:** Kristina N. Ekdahl, Karin Fromell, Marco Mannes, Karl‐Henrik Grinnemo, Markus Huber‐Lang, Yuji Teramura, Bo Nilsson

**Affiliations:** ^1^ Department of Immunology, Genetics and Pathology (IGP), Rudbeck Laboratory C5:3 Uppsala University Uppsala Sweden; ^2^ Linnæus Center of Biomaterials Chemistry Linnæus University Kalmar Sweden; ^3^ Institute for Clinical and Experimental Trauma‐Immunology University Hospital of Ulm Ulm Germany; ^4^ Department of Surgical Sciences, Division of Cardiothoracic Surgery Uppsala University, Uppsala University Hospital Uppsala Sweden; ^5^ Cellular and Molecular Biotechnology Research Institute (CMB), National Institute of Advanced Industrial Science and Technology (AIST) Tsukuba Japan; ^6^ Master's/Doctoral Program in Life Science Innovation (T‐LSI) University of Tsukuba Tsukuba Japan

**Keywords:** alternative pathway, complement regulation, extracorporeal treatment, hemodialysis, thromboinflammation

## Abstract

A number of clinical treatment modalities involve contact between blood and biomaterials: these include extracorporeal circuits such as hemodialysis, cardiopulmonary bypass, plasmapheresis, and intravascular treatments. Common side effects arising from these treatments are caused by activation of the cascade systems of the blood. Many of these side effects are mediated via the complement system, including thromboinflammatory reactions and rejection of implants. Depending on the composition of the materials, complement activation is triggered via all the activation pathways but is by far mostly driven by the alternative pathway amplification loop. On biomaterial surfaces the alternative pathway amplification is totally unregulated and leads under optimal conditions to deposition of complement fragments, mostly C3b, on the surface leading to a total masking of the underlying surface. In this review, we discuss the mechanism of the complement activation, clinical consequences of the activation, and potential strategies for therapeutic regulation of the activation, using hemodialysis as demonstrator.

## 1 INTRODUCTION

Medical treatments employing biomaterials in contact with blood usually trigger thromboinflammatory reactions immediately after blood contact which reduces the efficacy of the treatment and/or can cause harmful side effect to the patient. Examples of such treatment modalities include (nano)particles for drug delivery, different kinds of extracorporeal circuits (hemodialysis, hemofiltration, plasmapheresis, cardio‐pulmonary bypass [CPB], and extracorporeal membrane oxygenation [ECMO]) and intravascular devices cardiac aides, valves, and stents (reviewed in Ref. [1]).

**FIGURE 1:**
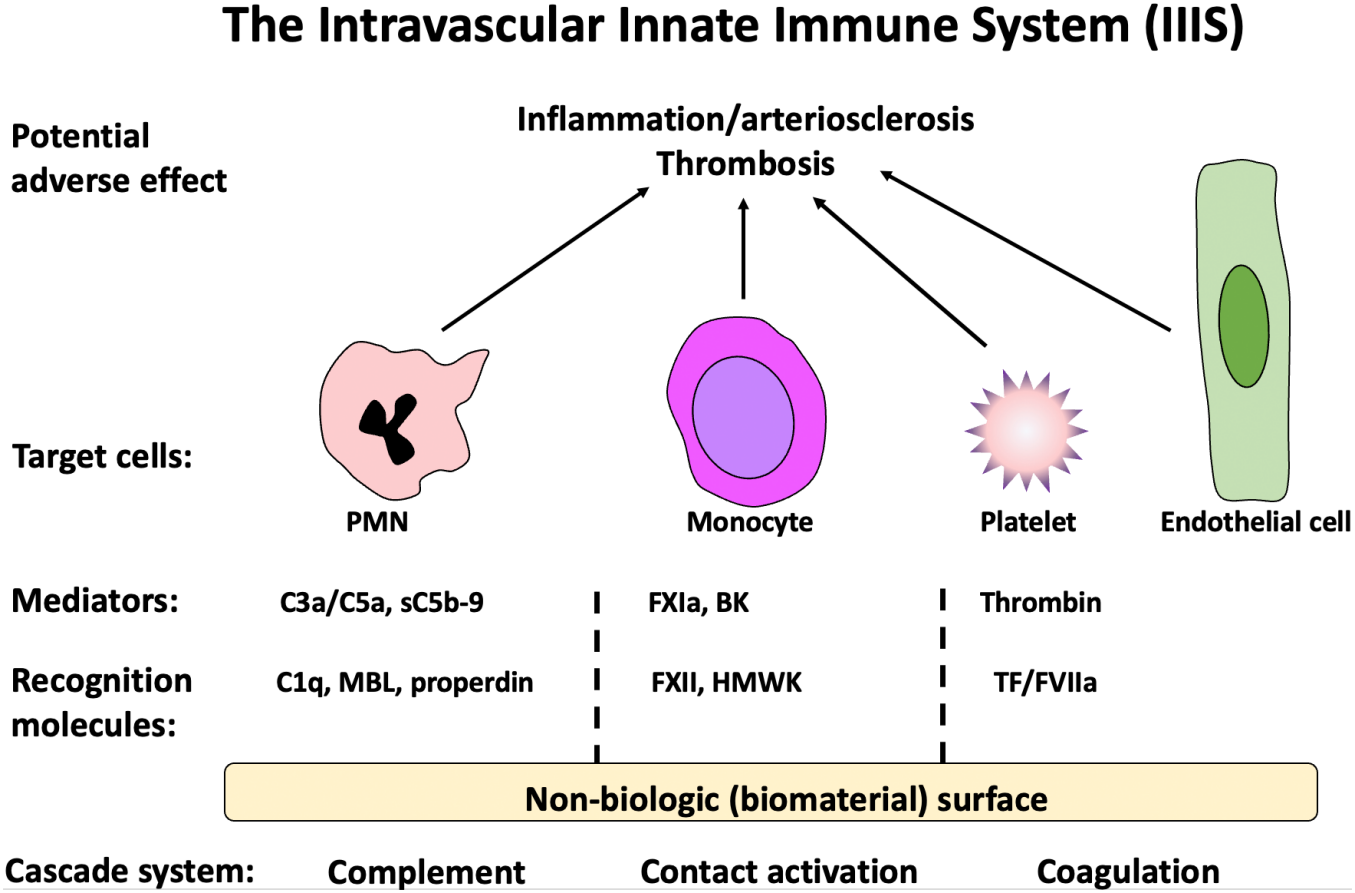
Intravascular Innate Immune System (IIIS). The IIIS is comprised of the blood plasma cascade systems (the complement, contact activation/coagulation, and fibrinolytic systems) and blood cells such as polymorphonuclear leukocytes (PMN), monocytes, platelets, and endothelial cells. Upon contact between blood and a non‐biological surface, for example, dialysis membrane, recognition molecules within the cascade systems target the surface initiating activation mechanisms and generate mediators, which in turn can bind and activate cells of the IIIS. Recognition molecules which have been implicated in recognition of artificial surfaces include C1q, MBL, and properdin in the complement system, Factor(F)XII and High Molecular Weight Kininogen (HMWK) in the contact activation system, and tissue factor (TF) and FVII of the coagulation system. Potent mediated are generated including the anaphylatoxins C3a and C5a, as well as the sC5b‐9 (complement), bradykinin (BK) and FXIa (contact activation), and thrombin (coagulation). Taken together, these reactions may result in a thrombo‐inflammatory reaction which may cause serious harm to the patient and/or the biomaterial. Reproduced from (ref. [78]) with permission from the publisher.

The thromboinflammation is elicited by the intravascular innate immunity system (IIIS), which consists of the blood cascade systems (the complement, contact activation/coagulation, and fibrinolytic systems) and blood cells such as platelets, polymorphonuclear leukocytes (PMN; granulocytes) and monocytes, and endothelial cells (Figure 1). Because IIIS activation basically is a surface‐oriented event, and due to cross‐talk between the humoral systems and cells, the thromboinflammation is instantaneously amplified upon contact between the blood and the medical device.^2^ In this review we will focus on the complement system (and in particular the alternative pathway), due to its central role in the bioincompatibility reactions that occur in these treatments.

## 2 THE COMPLEMENT SYSTEM

Approximately 50 proteins, present in the blood or bound to autologous cell surfaces are involved in the complement system, where they act as zymogens, receptors, or regulators. Complement has an important function for identifying and clearing pathogens and microorganisms, but also non‐biological compounds like man‐made biomaterials as “non‐self” in the body. Another vital function of complement is to distinguish and remove apoptotic and necrotic cells (both of which are “altered self”) from the healthy autologous cells (“self”). As soon as “non‐self” and “altered‐self” structures are recognized by the complement system on medical devices, the IIIS may trigger a destructive reaction leading to elimination of the devices and/or serious harm to the patient.

Complement is activated on biomaterial surfaces, and other non‐biological surfaces, via the three distinctive pathways: the classical pathway (CP), the lectin pathway (LP), and the alternative pathway (AP). All three pathways lead to the assembly of the CP/LP C3‐convertase complex, C4bC2a (according to the revised nomenclature C4bC2b^3^) and/or the AP C3‐convertase complex (C3bBb), both of which cleave C3 into the anaphylatoxin C3a and the opsonin C3b. Activation of the CP starts with the binding of the C1q component of the C1 complex to target‐bound antibodies, pentraxins, DNA, or other negatively charged substances. The LP is activated when the recognition molecules bind to carbohydrates, where the recognition molecules are the mannan‐binding lectin (MBL) or ficolins‐1, 2, and 3, as well as collectins 11/12, which are in complex with the MBL‐associated serine proteases (MASP)‐1 and MASP‐2. The surface activation of the CP and LP triggers proteolytic activation of C4 followed by C2 forming the C4bC2a convertase. When initiating C3b fragments are formed by any of the two pathways or by other, non‐canonical, mechanisms such as by non‐complement proteases, C3bBb convertases are generated supported by properdin, leading to the potent amplification loop of the AP. Additional C3b fragments associate with either of the two C3 convertases (ie, the CP/LP or the AP convertases) to form C5 convertases on the surface, where C5 is cleaved to the anaphylatoxin C5a and C5b. This cleavage is the first step in the terminal pathway, which ultimately forms the membrane attack complex (MAC), consisting of C5b, C6, C7, C8, and multiple copies of C9. Vitronectin is known to inhibit the MAC formation by suppressing C5b‐7 complex formation and C9 polymerization. In addition to the lytic function by the MAC formation, other essential effector functions of complement are the recruitment and activation of PMNs and monocytes to the material surfaces by the anaphylotoxins C3a and C5a. In addition, the surface bound opsonin C3b and its fragments iC3b and C3d,g facilitate phagocytosis of the target particle via interaction with complement receptors (CR1, CR3, CR4, and CRIg), which are upregulated on phagocytic cells in response to anaphylatoxins.

**FIGURE 2:**
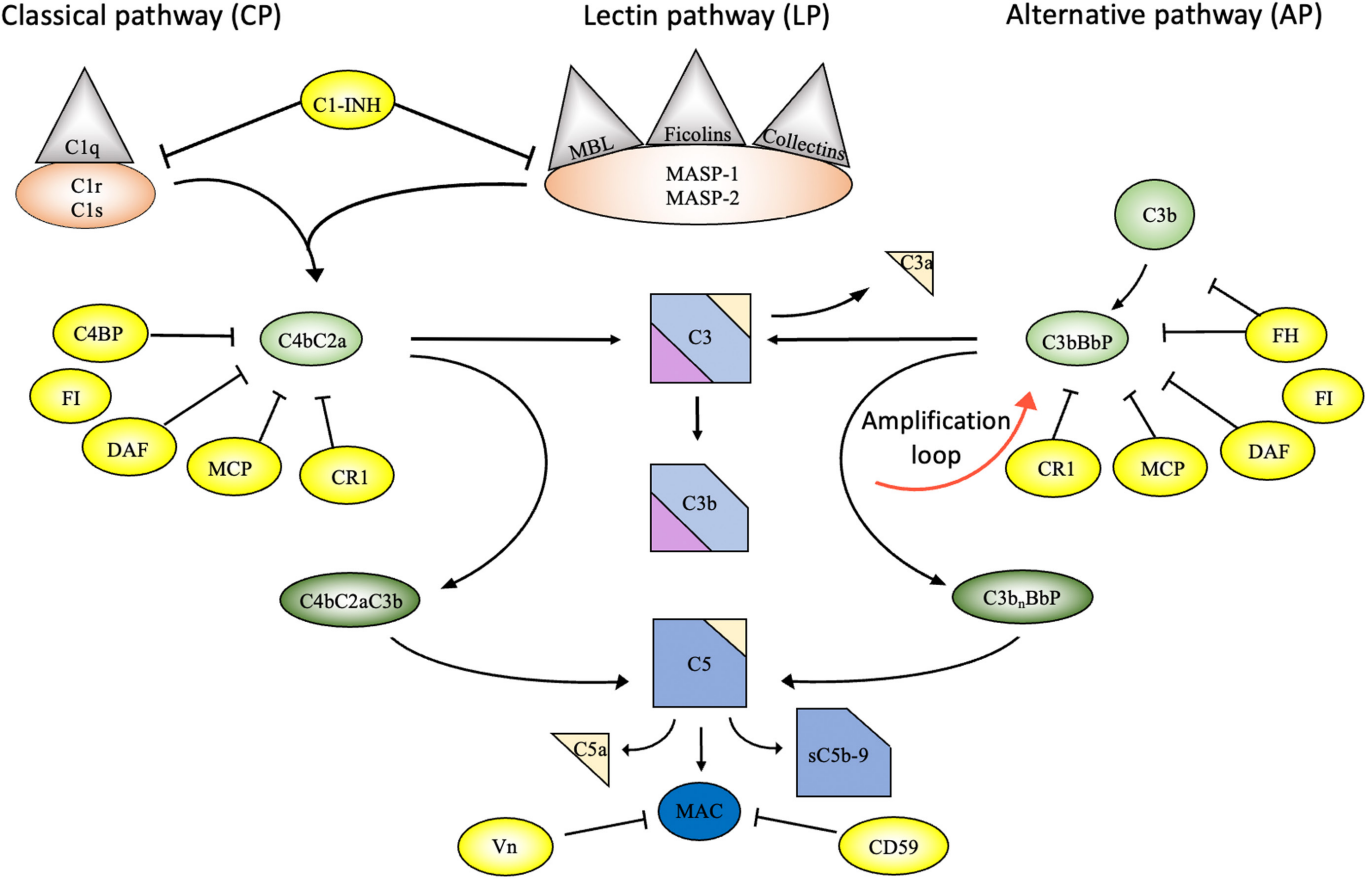
Overview of the complement system. Activation and regulation of the complement system (panel A). The complement system can be activated by three different pathways: the classical (CP), the lectin (LP), and the alternative (AP) pathways. Structures which are present on microorganisms but not on healthy autologous cells are targeted by recognition molecules within the pathways which leads to activation of the serine proteases C1r and C1s of the CP and MASP‐1, MASP‐2 of the LP. These proteases cleave and activate C4 and C2 to form the CP/LP C3‐convertase (C4bC2a). The convertases cleave C3 to the opsonin C3b and the anaphylatoxin C3a. The main role of the AP is to act as an amplification loop, due to the fact that each generated C3b is a potential nucleus of a new AP C3 convertase (C3bBbP). Deposition of C3b to either of the C3‐convertases convert them to C5‐convertases which cleaves C5 and thereby generates the anaphylatoxin C5a and initiates the assembly of the C5b‐9 complex, which may remain in the fluid phase as sC5b‐9 or become incorporated into the cell membrane in the form of membrane attack complex (MAC). Under physiological conditions, activation of complement activation is tightly controlled by a number of fluid phase and membrane inhibitors, most of which regulate the activity of the convertases. These include C4b‐binding protein (C4BP), decay acceleration factor (DAF, CD55), membrane cofactor protein (MCP, CD46), and complement receptor 1 (CR1, 35), factor H (FH), and factor I (FI). In addition, formation of the C5b‐9 complex is under control of CD59 and vitronectin (Vn), while the CP and LP serine proteases are inhibited by C1‐ inhibitor (C1‐INH). The figure is modified from Ref. [79] and published with open access under the Creative Commons CC‐BY license.

In the body, our autologous cells are protected from the attack of the complement system by numerous membrane‐bound and soluble regulators, so that our cells can be recognized and tolerated as “self” by the complement system. Most of the regulators are members of the “regulators of complement activation” (RCA) superfamily such as complement receptor 1 (CR1, CD35), decay acceleration factor (DAF; CD55), and membrane cofactor protein (MCP, CD46), which control the convertases. There are also soluble RCAs in plasma which include factor H and C4b‐binding protein (C4BP) which are regulators of the AP and CP, respectively. In addition to their fluid phase role, these soluble regulators can also be pulled down onto host cell surface by specific binding through glyocosaminoglycans, thereby regulating the convertases and protecting the cells against complement attack. The MAC is down‐regulated by CD59, which like DAF is bound to the cell membrane via a glycosylphosphatidylinisotol‐ (GPI) ‐anchor. Thus, all regulators are essential for avoiding thromboinflammatory attack against our autologous “self” cells. The organization, activation, and regulation of the complement system is summarized in Figure 2.

## 3 INTERACTION OF PLASMA PROTEINS WITH ARTIFICIAL MATERIAL SURFACES AND COMPLEMENT ACTIVATION

Activation of key complement components such as C3 at material surfaces can occur by proteolytic cleavage performed by complement proteases as well as by non‐canonical mechanisms such as proteolytic cleavage by non‐complement proteases and surface induced conformational changes.

### 3.1 Convertase mediated activation of C3

Protein adsorption onto artificial material surface occurs immediately when the materials come into contact with blood.^4^, ^5^ The plasma proteins instantly adsorb within seconds and form a monolayer on the material surface, creating a new interface between the material and the bulk of plasma proteins and blood cell populations. The composition and adsorbed amount of the protein layer strongly depend on the chemical and physical properties of the material surface.^6^, ^7^, ^8^, ^9^, ^10^ In particular, non‐target‐bound IgG^11^ and C3^12^ have been demonstrated in vitro to adsorb onto the surface from the plasma and change their conformation subsequently, leading to activation of the CP and the AP, respectively, while C1q, bound from serum was able to activate both the CP and AP.^13^ In addition, several studies demonstrate a selective depletion of ficolin‐2 during hemodialysis due to adsorption to in particular polysulfone membranes, suggesting an involvement of LP activation in hemodialysis‐induced inflammation.^14^, ^15^, ^16^ This effect is even more pronounced when heparin‐coated membranes are used and results in a quantitative loss of ficolin‐2.^17^

As complement activation proceeds, the nascent C3b molecule is able to specifically bind to proteins and carbohydrates in the first protein layer via free hydroxyl or amine groups, forming covalent ester and amide bonds, respectively.^18^ The binding of C3b to target surfaces via the covalent bond is essential for its biological activities due to, for example, its ability to activate the AP amplification loop to generate more C3b and its ability to act as ligand of complement receptors. On non‐biological surfaces, this C3 activation is mainly unregulated, since solid state material surfaces do not bind any of the physiological regulators such as factor H or C4BP, and activation can therefore continue in an unimpeded manner. This was evident in studies performed by Hed et al. on glass microscope slides where C3b deposition was followed by immunofluorescence and shown to continue until the whole slide was covered by C3b/iC3b.^19^ In later experiments, UR Nilsson showed that C3b molecules generated by the AP amplification loop eventually cover and mask initially bound proteins such as IgG and C1q, which thereby lose their function.^13^

The acceptor molecules for the covalent binding of C3b in the initial protein layer are largely unknown. Amide bonds binding C3b to proteins are stable from further nucleophilic attack. In contrast, ester bonds are more labile both due to the inherent esterase‐like activity of the bound C3b, and their sensitivity to nucleophilic attack by external agents such as NH_3_, and other amines, and even H_2_O, which may lead to spontaneous release of the deposited C3 fragments.^20^, ^21^, ^22^

Taken together, the initial C3 activation on a biomaterial surface can be triggered by all the pathways of complement, but subsequent activation is hi‐jacked by the uncontrolled AP amplification loop, since every generated C3b molecule is the potential nucleus of a new AP‐convertase complex (C3bBbP). Consequently, the quantitative role of the CP and the LP may be expected to be underestimated since the C3b subsequently generated by the AP amplification will cover and mask recognition molecules specific for the other two pathways (eg, C1q).

### 3.2 C3 conformational change‐induced activation

Native C3 binding to non‐biological surfaces in the initial protein layer is believed to be one of the initiators of the complement cascades on biomaterial surfaces. Native C3 bound to a plastic surface acquires the properties of C3b, possibly explained by a conformational change of C3 to C3(H_2_O) in that it can bind factor B and in the presence of factor D form a convertase that is able to cleave C3 to C3b and C3a.^12^ We have recently shown that fluid phase C3(H_2_O) generates an active but inefficient AP convertase.^23^ However, the formation of C3(H_2_O) leading to AP activation is likely to be greatly accelerated by the interaction with biomaterial surfaces. In a previous study, we demonstrated that C3 adsorbed to a flexible (soft‐branched structure) surface retained its native conformation while C3 adsorbed to a rigid (hard‐highly bonded) surface with the same general chemical structure was denatured exposing epitopes which are associated with binding of factor B and properdin, thereby having the potential to form a surface associated C3(H_2_O)‐containing AP convertase.^24^ In addition, surface‐bound C3(H_2_O) is less sensitive to regulation by factors I and H than surface‐bound C3b, suggesting that a C3(H_2_O)‐based AP convertase should have a longer half‐life, albeit with a lower specific activity compared with its C3b‐containing counterpart.

We have shown that a similar transformation occurs at a blood‐gas interface both in vitro when gas (air, O_2_ or N_2_ acting like an artificial surface) is bubbled through blood or plasma, and in a benchtop setup with a bubble oxygenator.^25^ C3(H_2_O) is also generated in vivo and can be detected in blood samples collected from cardiopulmonary bypass patients treated with bubble or membrane oxygenators.^26^ The generation of C3(H_2_O), elicits AP convertase activity and cleavage of native C3 into C3b and C3a in plasma, but does not proceed to sC5b‐9. Two conclusions can be drawn from this experiment: (1) High concentrations of C3(H_2_O) are needed in order for C3(H_2_O) to drive the AP; and (2) unlike solid‐phase materials, there are no C3b molecules present in the vicinity of the C3(H_2_O)Bb convertase that allows the formation of a C5‐convertase and assembly of sC5b‐9 complexes. Unlike the gas surface, sC5b‐9 formation on solid‐phase materials is a universal finding.

### 3.3 C3 convertase bypass mechanisms for C3b generation potentially operative at material surfaces

In addition to activation by the complement convertases, C3 has been demonstrated to be proteolytically digested by plasma membrane elastase and cathepsin G from U937 (monoblastoid) cells into C3a‐ and C3b‐like fragments.^27^ In addition, both C3 and C5 have also been reported to be cleaved by the coagulation proteases Factor (F)XIa, FXa, FIXa, and thrombin,^28^ as well as by plasmin^29^ to yield functional anaphylatoxins (C3a and C5a) and, in the case of plasmin, to also generate functional C5b‐9. Furthermore, cleavage of C5 by human leukocyte elastase has been demonstrated to generate of a functionally active C5b‐6 complex.^30^

Although all these observations were made in vitro using systems of purified proteases and complement proteins, it may be expected that these mechanisms are operative at or in close vicinity to biomaterial surfaces either (1) when leukocytes are bound and activated, thereby releasing their proteases or (2) when the coagulation cascade has been initiated at the surface generating the activated proteases, for example, FXa and thrombin. Leukocytes which fail to phagocytize a target due to large size (solid surface), undergo so called, “frustrated phagocytosis,” when they release a high concentration and variety of proteases.^31^ It should be pointed out that proteolytic cleavage of C3 and C5 by non‐complement proteases is not regulated by complement specific regulators: C4BP and factor I (CP/LP) and factor H and factor I (AP) which mainly control the activity of the C3 and C5 convertases.

## 4 ACTIVATION AND BINDING OF PLATELETS AND LEUKOCYTES TO MATERIAL SURFACES, SPREADING INFLAMMATION

**FIGURE 3:**
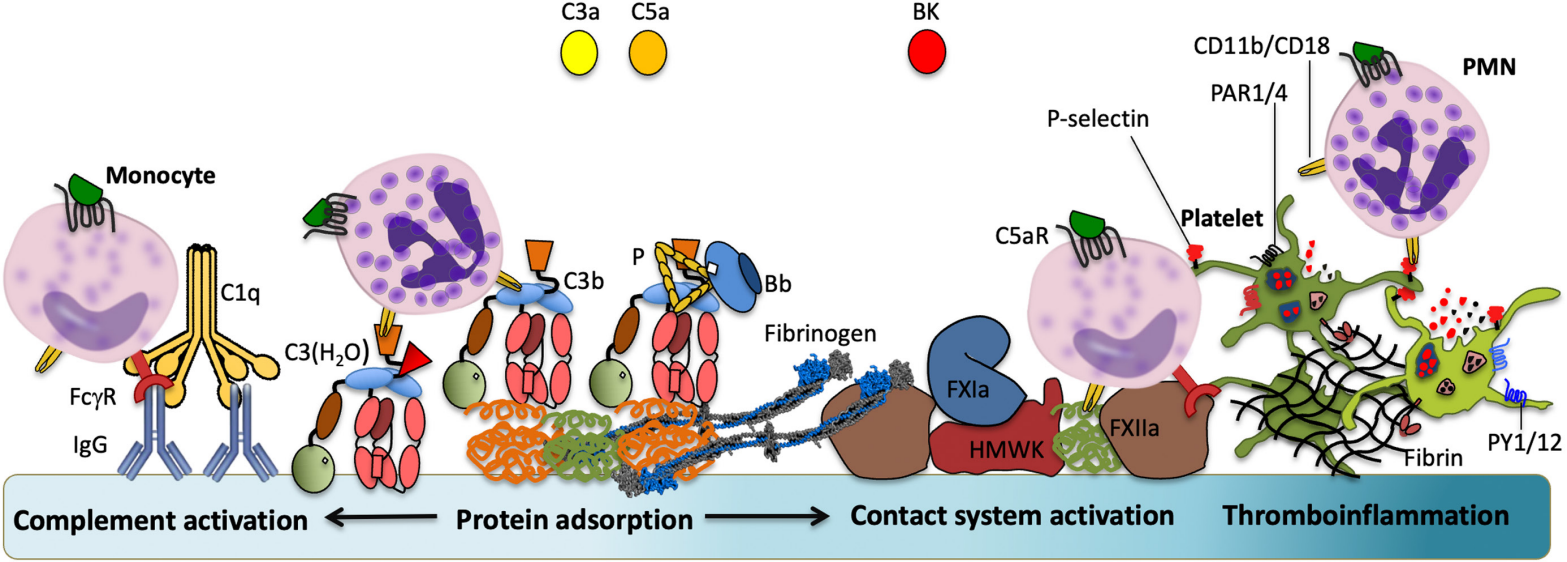
Protein adsorption and cascade system activation at material surfaces. When the surface of a biomaterial comes in contact with blood it will immediately become covered with a layer of plasma proteins by passive adsorption, approximately corresponding to a monolayer (schematically shown in green and orange). Recognition of the surface occurs by different mechanisms. Negatively charged surfaces have affinity for properdin and C1q which initiate complement activation via alternative pathway (AP) and the classical pathway (CP), respectively, as well as for Factor (F)XII which induces activation of the contact system. On hydrophilic surfaces, certain proteins bind in altered (“surface activated”) conformations. Proteins which are prone to bind in a denatured conformation resulting in subsequent activation include C3, which in the form of C3(H_2_O) activates the AP, and adsorbed (not bound to antigen) IgG, which activates the CP of complement. In addition, FXII binding in altered conformation will elicit contact system activation (via High Molecular Weight Kininogen [HMWK]) and coagulation activation (via FXIa). On top of the initial plasma protein film, complement (left) and contact system/coagulation (right) will be triggered and amplified, generating either anaphylatoxins (C3a and C5a) that recruit and activate leukocytes (polymorphonuclear cells [PMNs] and monocytes) or bradykinin (BK) thereby inducing an inflammatory response or thrombin that recruits platelets and catalyzes fibrin formation. Furthermore, during this process there is substantial cross‐talk between activated components within the different cascade systems, leading to further amplification of the inflammatory and pro‐thrombotic processes = thromboinflammation (far right).

The initial, passive, protein deposition on the material surface is followed by an active buildup of a thicker protein layer, mainly mediated by activation of the contact and complement systems (Figure 3). The bound proteins (eg, fibrinogen, fibrin, C3b, and iC3b) will provide ligands for platelets, PMNs, and monocytes and facilitate their binding to and activation on the surface. Complement activation produces the anaphylatoxins C3a and C5a as well as sC5b‐9 which are generated and released into the fluid phase from the surface^32^ and mediate leukocyte activation including upregulation of CD11b via C5a, whereby PMNs and monocytes are locally recruited by chemotaxis and bind to the artificial material surface, thereby causing a transient leukopenia.^33^, ^34^, ^35^

The leukocytes recognize material‐bound C3b/iC3b (and corresponding forms of C4‐fragments) via ligands such as CD11b/CD18 and CD11c/CD18 and get further activated by these interactions‐ This will trigger release of proinflammatory cytokines (IL‐1, TNF, IL‐6, IL‐8), elastase and metalloproteases, as well as up‐regulation and exposure of tissue factor (TF).^36^ TF will amplify the coagulation action via the extrinsic pathway.

Platelet activation accomplished by agonists such as thrombin and ADP, gives them the capacity to bind C3 in the form of C3(H_2_O).^37^, ^38^ Ultimately, these processes will lead to formation of complexes between activated platelets and PMNs and/or monocytes where C3(H_2_O) has been demonstrated to play a role.^38^ Activated platelet or platelet complexes bind to adsorbed fibrinogen or fibrin on the material surface via the integrin α_2_β_3._ ^39^

Hemodialysis is associated with complement activation and a rapid and transient leukopenia which mainly affects monocytes and PMNs, the levels of which reach a nadir around 15 min and are normalized within one hour.^35^ Massive generation of C3d,g can be detected in the plasma of dialysis patients^40^ where the peak levels coincide with the nadir of leukocytopenia.^41^ A likely explanation for this inverse relationship between peripheral cell counts and complement activation is that PMNs and monocytes adhere to the surface of the dialysis membrane as long as the deposited C3 remains in the C3b or iC3b forms which have high affinity for CD11b/CD18. Subsequently, when or if factor I in the plasma has cleaved iC3b to C3d,g, the cells may detach due to its low affinity for CD11b/CD18. In fluid phase and at physiological pH, plasma factor H is not able to act as a cofactor for factor I mediated digestion of C3‐fragments beyond iC3b.^42^ Therefore, the most likely co‐factor is CR1, either present on blood cells or in soluble form. CR1 has been demonstrated to be shed from erythrocytes by elastase derived from activated PMNs, in a form which is able to promote the cleavages of C3b/iC3b to C3d,g.^43^

In the dialysis setting, these highly activated leukocytes will follow the blood lines back to the patient, together with activated platelets, and proinflammatory mediators such as proinflammatory cytokines, bradykinin (BK), and the anaphylatoxins. Further activation of both platelets and leukocytes will be induced by the high shear force in the tubing. According to this model, the inflammation will spread from the initiating site at the material surface to the vascular tree of the patient, and thus promote systemic inflammation and contribute to endothelial activation, arteriosclerosis, and cardiovascular disease. This vicious circle of events is discussed in section 6.

## 5 SELECTED BIOMARKERS TO MONITOR COMPLEMENT ACTIVATION ON ARTIFICIAL MATERIAL SURFACES

Frequently used biomarkers to monitor complement activation induced by biomaterials include surface bound initiating molecules as well as activation products, either deposited on the surface or detected in the fluid phase.^44^

The binding of IgG, IgM, C1q to a surface may give a hint about the capacity to promote activation by the CP while ficolin 2 binding is associated with LP activation, and intact C3 or C3(H_2_O) may suggest ability to activate the AP. As a result of activation, C3 fragments (C3b, iC3b, and C3d,g) can be monitored on the surface, preferably by conformation specific mAbs,^45^ as well as activated C9 (contained in C5b‐9 complex) using neo‐mAb,^46^ which has become a very important antibody to detect sC5b‐9 in this type of diagnostics.

Following activation, C3a, C5a, and sC5b‐9 are all released into the fluid‐phase. C3a is a fairly stable and commonly used biomarker.^25^ In contrast, levels of C5a detected in the fluid phase may not reflect the true level of activation, since it binds with high affinity to C5aR:s which are found on leukocytes, and of which the level of expression is regulated by complement activation.^47^ Therefore, both fluid phase and leukocyte bound C5a need to be measured in order to assess complement activation at the C5‐level in vivo.^47^ Instead, it is more common to use the soluble terminal complex, sC5b‐9, as a surrogate marker for C5a generation. Furthermore, both C3a and C5a have high isoelectric point (pI ≈9) resulting in high positive charge at physiological pH, and they will therefore have affinity for dialysis membranes which normally are negatively charged, which may give falsely low levels of the anaphylatoxins in plasma. Furthermore, C3a and C5a (≈ 10 kDa) are small enough to be filtered out during dialysis using medium cut‐off membranes.^48^ In both cases, potentially unreliable results which do not visualize the true levels of complement activation in vivo, may be obtained when analyzing systemic blood samples from these patients.^49^

## 6 ADVERSE INNATE IMMUNE SYSTEM ACTIVATION AND THROMBOINFLAMMATION DUE TO BIOINCOMPATIBITY IN THE USE OF MEDICAL DEVICES

The quality of life has been improved due to the great progress of extracorporeal circulation including devices and artificial organs. Examples of such clinically available medical devices include hemodialysis, hemofiltration, ECMO, CPB, plasmapheresis, ventricular assistance devices, and valves all of which are composed of artificial materials such as synthetic polymers, ceramics, and metals.

During therapy, the surface of the device or vascular implant is continuously in contact with blood, and since no regulators of complement or coagulation are found on the artificial material surfaces, the blood cascade systems will be activated. The coagulation activation elicited by artificial materials is often induced by adsorption and activation of FXII, which leads to contact system activation and generation of the potent proinflammatory and pro‐angiogenic mediator BK.^50^ (Complement system activation, which is the focus of this article, was described in detail in sections 2 and 3.) These systems interact and collectively trigger a thromboinflammatory reaction at the material surface, where the proinflammatory mediators BK and the anaphylatoxins C3a and C5a are main players (Figure 3). Ultimately, the incompatibility‐elicited adverse reaction can spread to the whole body, leading to the death of the patient as a rare worst‐case scenario.

**FIGURE 4:**
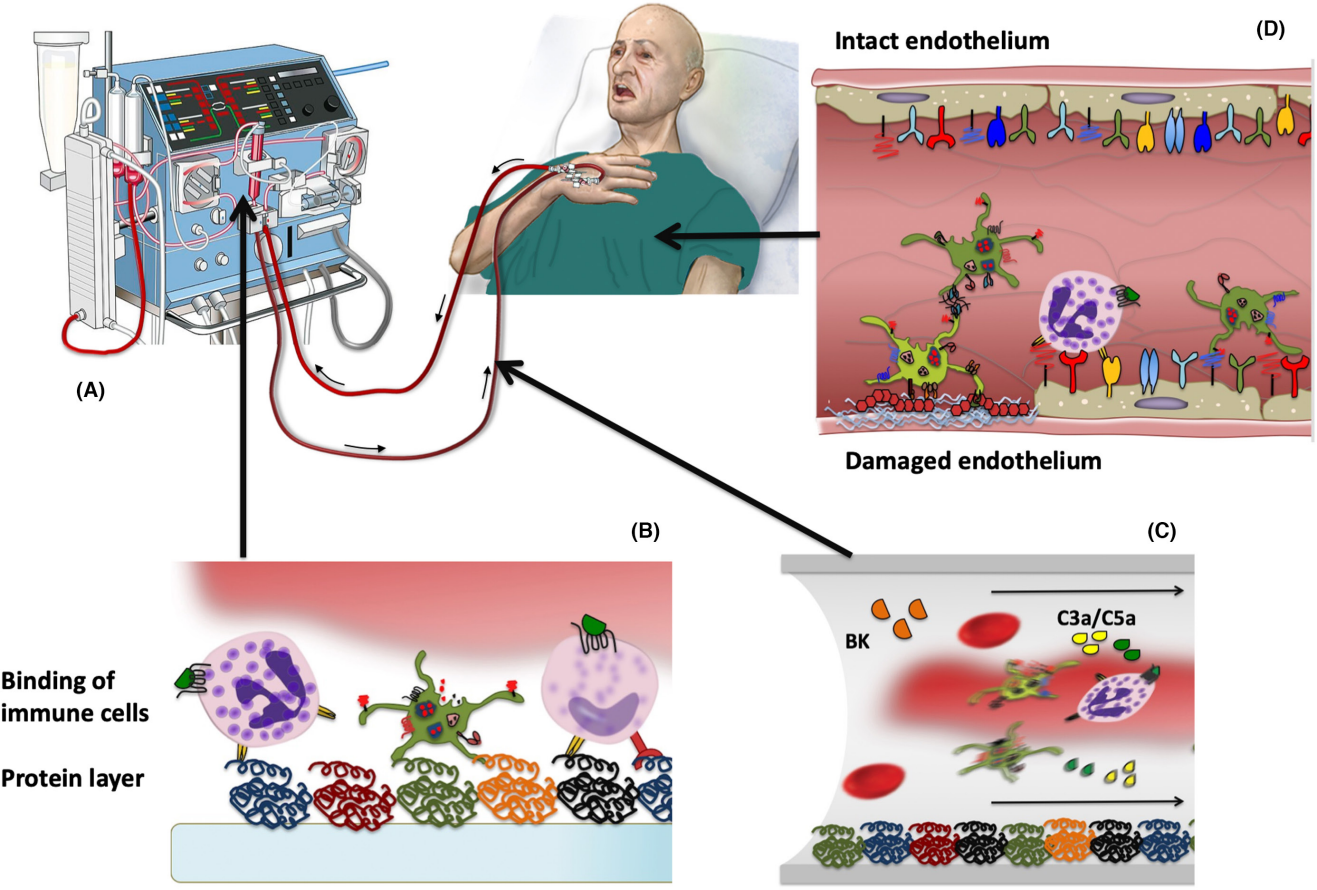
Model for innate immunity activation as a cause of cardiovascular disease initiated by hemodialysis. (A) During hemodialysis, the blood of the patient comes in prolonged and repeated contact with various tubing and dialysis membranes leading to activation of the intravascular innate immunity system (IIIS), in addition to activation taking place on the surface of gas bubbles produced by peristaltic pumps. (B) Proteins within the IIIS bind to the surfaces, triggering cascade system activation, recruitment and binding of platelets and leukocytes and resulting in clot formation and inflammation localized to the material surfaces (see also Figure 3). (C) Soluble mediators generated by the IIIS are together with activated platelets and leukocytes area transported back to the patient. Further activation of both cells and proteins will occur in the tubing due to the high shear force. (D) The patients' endothelium will be activated by these proinflammatory compounds, thereby losing its anti‐inflammatory and anti‐thrombotic properties, and be accessible for binding of activated leukocytes and platelets. These adverse reactions which occur during and after each dialysis session fuel a systemic inflammation and may contribute to the generation of arteriosclerosis and cardiovascular disease in the patient. Color coding: blue helices, collagen; red “string of pearls”, von Willebrand factor; blue dimers, bradykinin receptor 2 (BKR2). Reproduced from (ref. [78]) with permission from the publisher.

Although providing life‐saving support, these treatment options are associated with severe, potentially life‐threatening, clinical problems. For example, the risk of cardiovascular disease in hemodialysis patients is 5–10 times higher than that of healthy individuals.^51^ Typically, patients with end state renal disease receive hemodialysis treatment three times a week (several hours per session) for years. This leads to an ongoing chronic whole‐body inflammation since there are no hemodialysis membrane or extracorporeal tubing that are fully biocompatible, although significant improvements have been achieved over the years, and that treatment is performed under control by anticoagulants. The sequalae of events, initiated during hemodialysis which may ultimately lead to cardiovascular disease are summarized in Figure 4: the blood is pumped from the patient into the dialysis apparatus (Figure 4a), where it comes in contact with the dialysis membrane which typically is negatively charged thereby triggering IIIS activation (Figures 3 and 4b). In addition, gas bubbles, formed in the peristaltic pumps and collected in gas traps have the capacity to activate both the complement and contact system by presenting a hydrophobic interface between blood and gas on which molecules, for example, C3 and FXII can be denatured and activated. Activation products such as the anaphylatoxins C3a and C5a and BK are pumped back to the circulation of the patient at high velocity together with activated platelets and leukocytes. In addition, further activation of both proteins and cells occurs caused by the high shear force in these tubing (Figure 4c). During the process of intravascular inflammation, the patient's endothelium will be activated and lose its inherent anti‐thrombotic and anti‐inflammatory protection, leaving it vulnerable to binding of activated leukocytes and platelets, and contributing to arteriosclerosis and vascular disease in the patient (Figure 4d). There is a great number of publications describing damage on patient endothelium during and triggered by hemodialysis and aiming to dissect the underlying mechanisms, for example,^52^ and references therein.

Other examples of biomaterial‐related complications are that patients with vascular stents or heart valves are on anti‐platelet and anticoagulant drugs, respectively, in order to counteract the prothrombotic effects of these intravascular implants. Unless properly controlled, there is always a risk of bleeding, which can lead to deleterious effects.

## 7 SURFACE PROPERTIES AND MODIFICATIONS

The influence of surface‐bound functional groups on complement activation can be studied by using self‐assembled monolayers (SAMs) since they constitute a well‐defined surface. In such experiments the surface coated with SAMs carrying hydroxyl (OH), amino (NH_2_), methyl (CH_3_), and carboxyl (COOH) terminal groups is exposed to human serum or blood followed by detection of proteins on the surface by Surface Plasmon Resonance (SPR) using specific antibodies or by mass spectrometry.^8^ High amounts of complement proteins, particularly C3b has been found in the protein layer, indicating that complement activation through the AP has taken place. Interestingly, the adsorbed protein layer was almost totally composed of C3b and C3bBb and the binding of C1q, albumin and IgG was hardly detected on the OH groups. By contrast, deposition of C3b was hardly detected on the surface decorated with CH_3_, NH_2_, and COOH groups, indicating that those functional groups are less involved in the AP.^8^, ^53^, ^54^ Thus, surface functional groups can directly influence the complement activation as summarized in Table 1.

**TABLE 1:** Surface properties and modifications. Examples of influence of surface‐bound functional groups on binding of plasma proteins and complement activation on self‐assembled monolayers (SAMs) and novel polymers. Please see text (Section 7) for further details

Compound	Composition	Functional group	Properties at physiological pH	Binding of selected plasma proteins	Complement activation
**SAMS**					
SAM‐OH	11‐Mercapto‐1‐undecanol	OH	Hydrophilic	C3b (++) C1q (−), IgG (−), HSA (−)	High
SAM‐NH_2_	11‐Amino‐1‐undecanethiol	NH_3_^+^	Hydrophilic, cationic	C3b (+) C1q (−), IgG (−), HSA (++)	Moderate
SAM‐CH_3_	1‐Dodecanethiol	CH_3_	Hydrophobic	C3b (+) C1q (−), IgG (+), HSA (++)	Low
SAM‐COOH	11‐Mercaptoundecanoic acid	COO‐	Hydrophobic	C3b (+) C1q (+), IgG (−), HSA (+)	Moderate
**Polymers**					
P1	Methacrylic acid/N,N‐diacryloylpiperazine	COO‐	Hydrophilic, anionic	C3 (++), C4 (++), C1q (++), factor H (++) C4BP (++), IgG (++), HSA (+)	High
P2	Methacrylic acid/divinylbenzene	COO‐	Hydrophilic, anionic	C3 (+), C4 (−), C1q (−), factor H (+) C4BP (−), IgG (++), HSA (+)	Low
P3	Methacrylic acid/ethylene glycol dimethacrylate	COO‐	Hydrophilic, anionic	C3 (++), C4 (++), C1q (++), factor H (++) C4BP ++), IgG (++), HSA (++)	Low
P4	N‐Isopropyl acrylamide/ethylene glycol dimethacrylate	CONH_2_CH(CH_3_)_2_	Hydrophobic	C3 (−), C4 (+), C1q (−), factor H (−) C4BP (−), IgG (+), HSA (−)	Moderate
P5	Styrene/ethylene glycol dimethacrylate	C_6_H_5_	Hydrophobic	C3 (+), C4 (+), C1q (+), factor H (+) C4BP (+), IgG (+), HSA (+)	Moderate
P6	2‐Hydroxyethyl methacrylate/ethylene glycol dimethacrylate	OH	Hydrophobic	C3 (++), C4 (++), C1q (++), factor H (+) C4BP (+), IgG (+), HSA (+)	Moderate

*Note*: Binding score: (++), high; (+), moderate; (−) low/non‐detectable.

Abbreviations: C4BP, C4b‐binding protein; HSA, human serum albumin.

We have investigated the general properties of materials and the influence on the blood cascade systems by extensive characterization of six novel polymers (designated P1‐ P6, Table 1), together with three reference materials regarding, for example, composition, surface area, and pore size.^55^ Particles of these polymers were incubated in EDTA‐plasma, lepirudin‐plasma, or whole blood, followed by the detection of adsorbed proteins (20 different plasma proteins), activation markers of the complement and contact systems, and cytokine release. In this setting, we have for the first time proven a link between the initially adsorbed protein fingerprint and the inflammatory response in fresh human blood in contact with material surfaces. The C4/C4BP ratio (representing recognition and regulation of the complement system) and the FXII/C1‐INH ratio (representing recognition and regulation of the contact activation system) were shown to be reciprocally correlated. In addition, both ratios strongly correlated to the generation of 10, mainly pro‐inflammatory cytokines, including IL‐17, IFN‐γ, and IL‐6. Our conclusion was therefore that artificial surfaces in contact with blood either preferentially activate the complement system or the contact system, and that the profile of proteins adsorbed from EDTA‐plasma can serve as a useful surrogate marker for inflammatory response to materials intended for blood contact. By calculating 2 ratios (C4/C4BP, FXII/C1‐INH), we also applied the fingerprint pattern in a simple and robust method to predict hemocompatibility.^56^, ^57^ It should, however, be underscored that during prolonged contact, these initial recognition mechanisms will be overruled by amplification of C3b generation through the AP, leading to the majority of C3b deposition and anaphylatoxin generation, thereby orchestrating the adverse response against the biomaterial (see section 6. above).

## 8 DIFFERENT OPTIONS FOR ATTENUATION OF ADVERSE REACTIONS DURING TREATMENT WITH BIOMATERIALS

Potential therapeutic options to reduce the prevalence of adverse events during treatment with biomaterials ought to address the properties of the material itself, either its general physico‐chemical properties, or by different procedures for surface modification or functionalization. Systemic treatment with anticoagulants, for example, different forms of heparin, or platelet receptor antagonists are now common clinical practice, but the use of complement inhibitors is to a large extent still to be investigated. More specifically, standard pharmacologic treatment of patients with intravascular implants such as stents and heart valves is mainly focused on platelet inhibition and anticoagulation.^58^

### 8.1 Materials used in hemodialysis membranes and other applications with blood contact

Hemodialysis has been in clinical practice for many decades, and the hemocompatibility of the membranes has been significantly improved. Common materials in use today which include polysulfone, polyethersulfone, cellulose triacetate, polymethylmethacrylate (PMMA), ethylene vinyl alcohol copolymers, and polyacrylonitrile has enabled refined structures and lower immune activation.^59^ Most currently used membranes are composed of three layers of the synthetic polymers polyethersulphone/poly(vinylpyrrolidone)/polyamide where the outermost layer stabilizes the structure.^60^ These polymers are compatible with different solvents, and it is therefore possible to manufacture membranes with different pore size and thereby high permeability. The main drawback is that these membranes, as well as PMMA are hydrophobic and prone to adsorb and activate plasma proteins.^61^ Therefore, surface modification with poly(vinyl pyrrolidone) is used to increase the hydrophilicity and reduce clotting. In addition, other hydrophilic membranes with lower protein adsorption are available such as cellulose acetate and polyacrylonitrile.^62^ Other examples of materials frequently used in blood‐contacting equipment include segmented poly(ether urethane), used in artificial vessels, polytetrafluoroethylene (PTFE), poly(vinyl chloride) (PVC), both used in catheters, and poly(ether ketone) (PEEK) used in dental implants and bone fixation.^63^ In addition, various metals such as titanium and its alloys are being used in metallic devices.^64^

### 8.2 Coating biomaterials with the non‐biologic compounds, for example, MPC, PEGs, heparin

Two approaches for surface modification have been taken to avoid thromboinflammation caused by the hemoincompatibility: in both cases the surface of the medical devices is modified with functional polymers to regulate the interactions with biological events at the interface. One approach is to make the surface inert by inhibiting adsorption of plasma proteins and blood cells to the surface. To achieve this purpose, biocompatible polymers such as 2‐methacryloyloxyethyl phosphorylcholine (MPC)‐based polymer (MPC polymer)^65^, ^66^ and poly(ethylene glycol) (PEG)^67^, ^68^ have been successfully applied for coating of medical devices. Another approach is to fabricate bioactive surfaces by immobilizing heparin^26^, ^34^ on the material surface. In particular, the coating with MPC polymer and heparin have been shown to have the most potent non‐complement activation and anti‐thrombogenic property. Several types of N‐linked heparin coatings have been demonstrated to have superior non‐thrombogenic property, while coatings consisting of electrostatically bound heparin molecules have poorer function because the former coating provides freely moving heparin allowing a better antithrombin binding, while the latter one does not. Possible side‐effects are that the phospholipid containing MPC surface may bind C‐reactive protein (CRP).^69^ Also, heparin binds C1q to the negatively charged surface, but at the same time, complement activation is regulated by binding of factor H.^70^

### 8.3 Functionalization and/or auto‐protection of the biomaterial surface

Another approach to lower complement activation induced at a biomaterial surface is by conjugation of biologically active complement regulators, either naturally occurring or in an engineered form. We achieved proof of principle in two studies where we covalently bound human factor H purified from human plasma to model biomaterial surfaces using different conjugation chemistries. In both cases, we could demonstrate that complement activation was completely abrogated on the factor H‐decorated surfaces compared to non‐modified control surfaces emphasizing the importance of the AP amplification loop in the adverse complement activation on biomaterial surfaces.^32^, ^71^ However, despite these promising results obtained on a laboratory scale the approach to use full‐length proteins purified from human plasma is not practical for clinical use, because of the availability of human plasma for industrial applications, and since the procedure is costly and difficult to scale up. There is also the ever‐present concern of microbial contamination^72^ as well as the batch‐to‐batch variation when human plasma is used as raw material for purification.

Instead, we are currently following a more targeted approach using synthetic peptides with affinity for soluble complement regulators to pull down the protein in question (factor H or C4BP) from the plasma of the patient, thereby providing protection with endogenous proteins and avoiding the issues mentioned above. Factor H binding peptides were identified by using a cysteine‐constrained phage‐displayed peptide library. From these screening experiments, the peptide 5C6 which bound to the broad middle region of factor H (SCR 5–18) with high affinity, was selected for further studies. When immobilized on polystyrene (model biomaterial) surfaces using biotin‐streptavidin, it captured factor H from plasma and significantly inhibited complement activation in undiluted plasma by the AP.^73^ We also used peptide 5C6 in conjunction with an ADP‐degrading enzyme, apyrase, in order to inhibit platelet aggregation at the material surface in addition to complement activation. This hybrid surface provides a promising approach because it was able to substantially inhibit both complement and coagulation at the surface in a xeno‐transplantation model, indicating that the function of each ligand (the peptide 5C6 and the enzyme apyrase) remained intact.^74^

### 8.4 Systemic complement inhibition during biomaterial‐based treatments

In the last decades, a number of inhibitors of complement activation has been developed. Most of them are recombinant proteins and homologous to natural physiological inhibitors. In later years, small‐molecular and RNA‐based inhibitors have also been produced. The first inhibitor that was commercially available was a soluble form of the membrane‐bound complement receptor 1 (CD35, TP‐10), which was tested in a number of clinical situations. Despite the fact that promising results were obtained in a biomaterial associated trial of safety and efficacy of TP10 in adult women undergoing cardiopulmonary bypass surgery,^75^ this substance never made it to the market. The majority of complement inhibitors produced until now belongs to three major groups targeting either the C1‐complex, the AP or C5. The first licensed drugs were C1INH enriched from plasma (Beherinert®) and recombinant anti‐C5 (eculizumab). C1INH preparations inhibit the C1‐complex at the C1r and C1s subunits and MASP‐1 and MASP‐2, while eculizumab blocks cleavage of C5 into C5a and C5b. Eculizumab is approved as an orphan drug for paroxysmal nocturnal hemoglobinuria (PNH), atypical hemolytic uremic syndrome (aHUS), myasthenia gravis (MG), and neuromyelitis optica spectrum disorder (NMOSD), and has also been used off‐label in several other conditions.

The aim of using systemic complement inhibitors in blood‐contacting biomaterial‐based treatment modalities is to reduce thromboinflammation and thereby avoid biomaterial induced disorders and side effects. There are many newly developed inhibitors that potentially may be used to regulate complement activation associated with blood in contact with a biomaterial surface, but this depends on where in the complement cascade that the drug is targeting. In order to do so, a reduction of both surface (binding of C3 fragments) and fluid‐phase activation (generation of anaphylatoxins and other activation products) must be achieved.

To reach this inhibition profile, blocking of C3 and up‐stream must be accomplished, since cleavage of C3 is obtained already by the CP/LP convertase. Taken all aspects into account, short‐acting C3 inhibition is likely to be most efficient and with the lowest risk of side effects. Candidates are peptides from the compstatin family or inhibitory antibodies. It will block the CP and AP convertases, deposition of C3 fragments on the material surface and also the generation of anaphylatoxins, and other activation products such as sC5b‐9.

Pure AP inhibitors such as factors B and D inhibitors might not block C3b binding completely, since the CP/LP convertase is still active, and inhibition of C5 by eculizumab and other C5/C5aR1 inhibitors would still allow activation of C3 with C3b deposition on the material surface. Eculizimab (anti‐C5) has been tested in vitro in whole‐blood models and been shown to deplete the material surface of granulocytes and monocytes after exposure to whole blood. This binding is mediated by CD11b/CD18, which is upregulated by C5a. However, in vivo several other agents regulate this receptor, for example, BK, which allows binding of granulocytes and monocytes to the material surface where C3 fragments will still be available. An additional alternative would be C1INH or a C1s inhibitor, but also here the AP would be active. C1INH is not a specific complement inhibitor since it also blocks FXIIa, FXIa, and kallikrein of the contact system, potentially still making it a valuable thromboinflammatory inhibitor, but no studies have been performed using these C1‐related drugs on a blood‐contacting indication.

### 8.5 Application of soluble inhibitors tried in hemodialysis models

C3 inhibition can be obtained using mAbs, RNA‐based drugs, small inhibitory molecules, or blocking peptides (the compstatin family). Compstatin, a C3 inhibiting circular peptide, was early tested in vitro for use with blood‐contacting biomaterials. In a whole‐blood loop test in vitro, it was shown that compstatin inhibited complement activation triggered by both the material surface and the gas‐blood interface. Both the generation of fluid phase activation products such as C3a and sC5b‐9 and surface‐bound C3 fragments were inhibited. As a consequence of this, granulocytes and monocytes did not bind to the surface, leaving only platelets sticking to the material surface. Kourtzeliz et al. and Reis et al. showed in bench top hemodialysis systems in vitro that complement was inhibited, and that side effects induced by the hemodialyzer such as the expression of MCP‐1, CD11b, and TF, were inhibited, that the anti‐inflammatory cytokine IL‐10 was upregulated, thereby collectively inhibiting clotting and cytokine induced inflammation.^36^, ^76^ The recent version of unconjugated compstatin, such as AMY‐101, has a very high affinity to C3 making it even more suitable for hemodialyisis since almost no substance appears in the free form but only in complex with native C3. This property avoids the risk that the peptide is passing through the dialysis membrane, (thereby decreasing the available concentration and hence its inhibitory effect), which is always the risk with a low molecular weight substance.^48^ Recently, pegcetacoplan was licensed for PNH.^77^ This is a PEGylated peptide of the compstatin family and represents the third type of anti‐complement drug available at the market.

## 9 CONCLUSION

Side effects arising from treatment modalities involve contact between blood and biomaterials in treatments involving extracorporeal circuits and intravascular devices. These are arising from the cascade systems of the blood, mostly mediated via the complement system and involve thromboinflammatory reactions that rejects biomaterial. On biomaterial surfaces, the AP amplification is totally unregulated and leads under optimal conditions to deposition of complement fragments, mostly C3b, on the surface leading to a total masking of the underlying surface that drives the adverse reaction.

## FUNDING INFORMATION

The work described in this review article was supported by the Swedish Foundation for Strategic Research; project SBE13‐0028 “Strategies for stem cell survival”; the Swedish Research Council (VR) grants 2016–01060, 2016–04519, 2020–05762, 2021–02252; the Swedish Foundation for International Cooperation in Research and Higher Education (STINT); the Bilateral Joint Research Projects (Japan‐ Sweden) of the Japan Society for the Promotion of Science (JSPS); Grant‐in‐Aid for Scientific Research for Fostering Joint International Research (18KK0305) from the Ministry of Education Culture Sports Science and Technology (MEXT) of Japan; faculty grants from the Linnæus University.

## CONFLICTS OF INTEREST

The authors declare no conflicts of interest.

## Data Availability

Data are available on request from the corresponding author.
